# The Top 100 Highly Cited Original Articles on Immunotherapy for Childhood Leukemia

**DOI:** 10.3389/fphar.2019.01100

**Published:** 2019-09-24

**Authors:** Qing Zhong, Bing-Hui Li, Qi-Qi Zhu, Zhi-Min Zhang, Zhi-Hao Zou, Ying-Hui Jin

**Affiliations:** ^1^Department of Pediatrics, Guangzhou Hospital of Integrated Traditional and West Medicine, Guangzhou, China; ^2^Center for Evidence-Based and Translational Medicine, Zhongnan Hospital of Wuhan University, Wuhan, China; ^3^Center for Evidence-Based Medicine, Institute of Evidence-Based Medicine and Knowledge Translation, Henan University, Kaifeng, China

**Keywords:** childhood leukemia, immunotherapy, bibliometrics, Web of Science, VOSviewer

## Abstract

**Background:** Childhood leukemia is one of the most common cancers in children. As a potential treatment for leukemia, immunotherapy has become a new research hotspot. This research aimed at exploring the status and trends of current researches on immunotherapy for childhood leukemia through bibliometric analysis.

**Methods:** The Institute for Scientific Information Web of Science core collection database was searched for articles on immunotherapy and childhood leukemia using a computer. Time period for retrieval was from the beginning of the database to June 15, 2019. The top 100 highly cited articles were selected to extract their information on publication year, authors, title, publication journal, number of citations, author’s affiliations, country, and so on. These general information and bibliometric data were collected for analysis. VOSviewer software was used to generate a figure for keywords’ co-occurrence network and a figure for researcher’s coauthorship network that visualized reference and cooperation patterns for different terms in the 100 articles.

**Results:** The number of citations in the top 100 articles ranged from 17 to 471. These articles were published in 52 different publications. The top four journals in terms of the number of our selected articles were *Leukemia* (11 articles), *Blood* (10 articles), *Bone Marrow Transplantation* (6 articles), and *Clinical Cancer Research*. The most frequently nominated author was T. Klingebiel from Goethe University Frankfurt, and of the top 100 articles, 12 listed his name. These top 100 articles were published after the year 2000. Most of these articles were original (67%). The United States and Germany were the major countries researching immunotherapy for childhood leukemia and made significant contributions to the combat against the disease. Adoptive immunotherapy and stem cell transplantation appeared more frequently in keywords.

**Conclusions:** This study analyzed the top 100 highly cited articles on immunotherapy for childhood leukemia and provided insights into the features and research hotspots of the articles on this issue.

## Introduction

Leukemia is a malignant clonal disease of hematopoietic stem cells (Greaves, 2016). It is estimated that this disease will see more than 0.4 million new cases and 0.3 million related deaths worldwide, according to the GLOBOCAN 2018 (Bray et al., 2018). And new leukemia cases account for 31% of new patients of childhood malignancies (Ward et al., 2014). Most children with leukemia show rapid onset (Miranda-Filho et al., 2018). Due to the complexity in leukemia typing and prognosis, there is no one-size-fits-all treatment for the disease (Pui et al., 2015; Lam et al., 2017). At present, main treatment methods for the disease contain the following types: chemotherapy (Saygin and Carraway, 2017), radiation therapy (Simone et al., 2012), targeted therapy (Mugoni et al., 2019), immunotherapy (Acheampong et al., 2018), stem cell transplantation (Cornelissen and Blaise, 2016), and the like. After reasonable and comprehensive treatment, the prognosis of leukemia has been greatly improved (Marcos-Gragera et al., 2017). A considerable number of patients can be cured or reach long-term stability (Pui et al., 2003; Lennmyr et al., 2019).

Immunotherapy, a new treatment for cancer, can help the immune system fight cancer (Majzner et al., 2017). Over the past few decades, immunotherapy has developed targeting cancer at a striking rate (Foster and Maude, 2018). There are many immunotherapies for leukemia, such as chimeric antigen receptor (CAR) T-cell therapy, bispecific T-cell engager (BiTE) therapy, and antibody-drug conjugates (ADCs) (Aldoss et al., 2017; June et al., 2018; Liu et al., 2019). Chimeric antigen receptor T-cell technology is the most influential immunotherapy for childhood leukemia in the last decade (Gardner et al., 2017). This measure uses autologous T cells to attack malignant cells (Wang et al., 2017). Studies have shown that CAR T cells are effective in inducing remission among leukemia patients and thus provide valuable opportunities for subsequent transplantation, finally achieving durable remission (Pan et al., 2019). Evidence also has shown that CAR–T-cell therapy can achieve fine effects among patients with recurrent B-cell malignancies or those facing relapse after cord blood transplant, with fewer complications (Fan et al., 2017). Bispecific T-cell engager therapy is also a new advance in immunotherapy for childhood leukemia. Preclinical studies have shown that BiTE can realize antileukemia function by targeting T cells and CD33^+^ monocyte myelogenous suppressor cells, recruiting and expanding autologous T cells and inducing acute myeloid leukemia -blasts lysis (Krupka et al., 2016; Jitschin et al., 2018). Antibody-drug conjugate therapy, another immunotherapy, has been widely concerned. In this approach, cytotoxic molecules could bind to antibodies, and then the antibodies specifically bind to specific tumor antigens, and the cytotoxic molecules would be endocytosed into cells, thereby killing tumor cell from inside (Foster and Maude, 2018). An *in vitro* experiment showed that ADCs could improve the antiproliferation and cytotoxicity of human acute lymphoblastic leukemia cell lines (Hicks et al., 2019). In the mouse model of leukemia, ADC treatment could significantly improve survival rate without overt toxicity or adverse effects (McGinn et al., 2017). As an effective antileukemia immunotherapy, ADC has been assessed for its safety and efficacy in leukemia patients (Li et al., 2018).

Immunotherapy is important in treating childhood leukemia, but there is no bibliometric analysis on researches in this field. The purpose of this study was to use bibliometric methods to analyze the top 100 highly cited articles on immunotherapy for childhood leukemia, hoping to have a better understanding of current situation and trend of those researches through analyzing their main characteristics.

## Materials and Methods

### Data Sources

Literature on immunotherapy for childhood leukemia was retrieved from Institute for Scientific Information (ISI) website of the Science Core Collection Database of Henan University from the beginning of the database to April 30, 2019 (updated to June 15, 2019). *Childhood*, *pediatric*, *leukemia*, and *immunotherapy* were used as search terms. Retrieved documents were arranged in descending order according to the number of citations, and the top 100 most cited articles were finally obtained.

### Data Extraction

The top 100 most frequently cited articles were selected, and the following information was extracted from them: the number of citations, the names of the authors, authors’ affiliations, country, publication year, article title, article type, journal, Web of Science categories, quartile in category, and impact factor of the journal (2017 edition of Journal Citation Reports).

Two independent researchers evaluated each identified article to identify articles involving immunotherapy for childhood leukemia, regardless of article type. If there were different opinions, a third reviewer would be consulted, and consensus was thus achieved through discussion.

### Statistical Analysis

Microsoft Excel 2013 software was used for descriptive statistical analyses, including those on publication year, author, author affiliation, country, journal, and citation number. VOSviewer 1.6.8 (van Eck and Waltman, 2010) was used to draw figures for keyword co-occurrence network and coauthored network, so as to implement network visualization analysis. In network visualization, each circle and label represented a keyword or researcher, and the size of circles represented the frequency of occurrence. The larger the circle was, more frequently the circle-represented body appeared. Circles adopting different colors in graph represented different clusters. Lines between two circles indicated that two keywords or researchers appeared together. The thicker the lines were, more frequently they appeared together. More relevant two keywords or researchers were, closer two circles located. The minimum number of co-occurrences was adjusted according to graphic results.

## Results

### Characteristics of Included Studies

**Figure 1:**
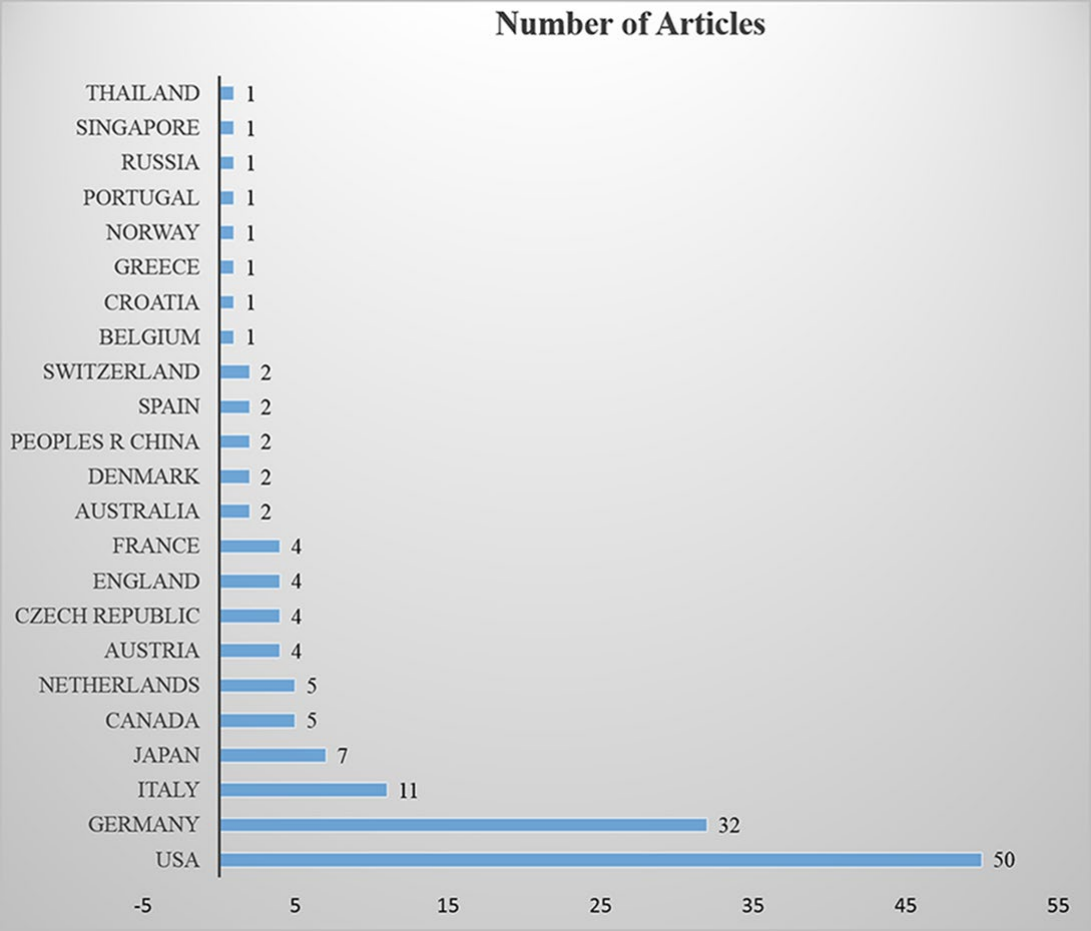
Countries from which the 100 most highly cited articles originated and the number of our collected articles from each of those countries.

A total of 360 articles were retrieved from the Web of Science Core Collection Database to introduce immunotherapy for childhood leukemia. Articles are listed in descending order according to the cited frequency; the top 100 articles with the highest cited frequency are selected. All of the top 100 highly cited articles were published between 2000 and 2018, about two to nine articles each year. Of the articles, 99 were published in journals and 1 in a book. These articles were in 19 categories, including hematology, oncology, and immunology. The 100 articles were published by researchers from 23 countries, most from the United States, Germany, Italy, and Japan (**Figure 1**). The 100 articles were published in 52 publications, 7 of which were not included in the 2017 edition of Journal Citation Reports. The quartile in category was distributed in Q1–Q4; impact factors of the journals ranged from 0.698 to 32.621, and basic information of the journals is shown in **Table 1**. The average and median number of citations, range of citation number, and interquartile range of the 100 articles were 53.2, 35.5, 17 to 471, and 35.25, respectively. The articles contained 67 original articles, 23 reviews, 6 conference abstracts, 2 letters, 1 editorial material, and 1 book chapter. The information of the top 100 highly cited articles is listed in **Supplementary Table S1**.

**Table 1:** Journals publishing the top 100 most highly cited articles^#^.

Journal	No. of articles	Quartile in category	Impact factor	Citation count	Country
*Leukemia*	11	Q1	10.023	487	England
*Blood*	10	Q1	15.132	495	USA
*Bone Marrow Transplantation*	6	Q1	4.497	257	England
*Clinical Cancer Research*	6	Q1	10.199	364	USA
*British Journal of Haematology*	4	Q1	5.128	134	USA
*Biology of Blood And Marrow Transplantation*	3	Q1	4.484	97	USA
*Journal of Clinical Oncology*	3	Q1	26.36	570	USA
*Klinische Padiatrie*	3	Q4	0.698	126	Germany
*Blood Cells Molecules and Diseases*	2	Q4	1.836	175	USA
*Cancer Genetics and Cytogenetics*	2	—	—	134	USA
*Cancer Research*	2	Q1	9.13	74	USA
*Haematologica—The Hematology Journal*	2	—	—	67	Italy
*International Journal of Hematology*	2	Q3	1.942	170	USA
*Journal of Immunology*	2	Q2	4.539	54	USA
*Medical and Pediatric Oncology*	2	—	—	231	USA
*Pediatric Blood & Cancer*	2	Q1	2.646	74	USA
*PLoS One*	2	Q1	2.766	74	USA
*Therapeutic Advances in Hematology*	2	—	—	81	England
*Angewandte Chemie—International Edition*	1	Q1	12.102	34	Germany
*Annals of Oncology*	1	Q1	13.93	28	England
*Annual Review of Medicine*	1	Q1	14.97	197	USA
*Arthritis and Rheumatism*	1	—	—	151	USA
*Biomarkers*	1	Q3	1.976	40	England
*Cancer Cell*	1	Q1	22.844	30	USA
*Cancer Discovery*	1	Q1	24.373	251	USA
*Cancer Immunology Immunotherapy*	1	Q2	4.225	21	USA
*Cancer Journal*	1	Q2	3.519	17	USA
*Cancer Journal from Scientific American*	1	—	—	18	USA
*Clinical Immunology*	1	Q2	3.557	20	USA
*Current Medicinal Chemistry*	1	Q2	3.469	19	United Arab Emirates
*Current Opinion in Hematology*	1	Q2	2.821	19	USA
*Current Opinion in Immunology*	1	Q1	7.932	89	England
*Cytotherapy*	1	Q1	3.993	19	England
*Discovery Medicine*	1	Q3	2.398	24	USA
*Drug Discovery Today*	1	Q1	6.848	25	England
*Expert Review Of Hematology*	1	Q3	1.937	26	England
*Frontiers in Immunology*	1	Q1	5.511	20	Switzerland
*Frontiers in Pediatrics*	1	Q2	2.335	27	Switzerland
*Haematologica*	1	Q1	9.09	20	Italy
*Immunological Reviews*	1	Q1	9.217	36	USA
*Immunology Letters*	1	Q3	2.436	51	Netherlands
*International Journal of Cancer*	1	Q1	7.36	26	USA
*Journal of Allergy and Clinical Immunology*	1	Q1	13.258	80	USA
*Journal of Oncology Pharmacy Practice*	1	Q3	1.908	17	England
*Leukemia Research*	1	Q3	2.319	19	England
*Molecular Immunology*	1	Q2	3.188	20	England
*Nature Medicine*	1	Q1	32.621	123	USA
*Nature Reviews Clinical Oncology*	1	Q1	24.653	52	USA
*Oncologist*	1	Q1	5.306	19	USA
*Oncotarget*	1	—	—	31	USA
*Pediatric Hematology and Oncology*	1	Q3	1.154	23	USA
*Science Translational Medicine*	1	Q1	16.71	64	USA

^#^Data from the 2017 edition of Journal Citation Reports.

### The Top 10 Authors

Among the 100 articles, the researchers who published most articles were T. Klingebiel from Goethe University Frankfurt, Germany, reaching a total of 12 articles, while the second and third authors came from the same university. Of the top 10 authors, nine were from Germany, four from the United States, three from Japan, and one from Italy (**Table 2**).

**Table 2:** Top 10 authors most frequently appearing in the articles.

Rank	Author	Number of articles	Affiliation	Country
1	Klingebiel T	12	Goethe University Frankfurt	Germany
2	Bader P	10	Goethe University Frankfurt	Germany
3	Lang P	8	Eberhard Karls University of Tubingen	Germany
4a	Gruhn B	6	Friedrich Schiller University of Jena	Germany
4b	Grupp Sa	6	University of Pennsylvania	USA
4c	Handgretinger R	6	Eberhard Karls University of Tubingen	Germany
4d	Niethammer D	6	Osaka University	Japan
4e	Sugiyama H	6	Osaka University	Japan
9	Kreyenberg H	5	Goethe University Frankfurt	Germany
10a	Barrett Dm	4	University of Pennsylvania	USA
10b	Brown P	4	Johns Hopkins University	USA
10c	Dilloo D	4	Heinrich Heine University Dusseldorf	Germany
10d	Kremens B	4	University of Duisburg Essen	Germany
10e	Locatelli F	4	University of Pavia	Italy
10f	Mackall Cl	4	Stanford University	USA
10g	Oka Y	4	Osaka University	Japan
10h	Zintl F	4	Friedrich Schiller University of Jena	Germany

### The Top 10 Institutions

Like Eberhard Karls University of Tubingen and National Institutes of Health, the University of Pennsylvania, produced 10% of the top 100 articles, followed by the Goethe University Frankfurt (8%), Johns Hopkins University (8%), and National Cancer Institute (8%) (**Table 3**).

**Table 3:** Institutions contributing to the 100 most highly cited articles.

Institution name	Country	Number of articles
Eberhard Karls University of Tubingen	Germany	10
National Institutes of Health	USA	10
University of Pennsylvania	USA	10
Goethe University Frankfurt	Germany	8
Johns Hopkins University	USA	8
NIH National Cancer Institute	USA	8
Children’s Hospital of Philadelphia	USA	7
Johns Hopkins Medicine	USA	7
St. Jude Children’s Research Hospital	USA	7
Osaka University	Japan	6

### The Top 10 Articles

The top 10 highly cited articles contained four original articles, five reviews, and one book chapter (**Table 4**). They were from the United States, Italy, Germany, Japan, and Australia. Of the 10 articles, 6 were published in journals, which ranked Q1 in the 2017 edition of Journal Citation Reports, and 1 in that ranking Q3, while journals publishing the other 3 were not included in the 2017 edition of Journal Citation Reports. The categories of these journals involved blood, tumors, pediatrics, and the like. The top 10 articles covered the updating of treatments for childhood leukemia (Rodriguez-Galindo et al., 2003; Pui et al., 2011; Locatelli et al., 2012; Barrett et al., 2014), different mechanisms of immunotherapy (Sotillo et al., 2015; Fry et al., 2018), WT1 targeted therapy (Sugiyama, 2001; Rosenfeld et al., 2003), a case series report (Diak et al., 2010), and a clinical study (Dagher et al., 2002).

**Table 4:** Top 10 most highly cited articles^$^.

Author	Title	Year	Journal	Quartile in category	Impact factor	Citation count	Article type	Country
Pui CH	Biology, risk stratification, and therapy of pediatric acute leukemias: an update	2011	*Journal of Clinical Oncology*	Q1	26.36	471	Review	USA
Sotillo E	Convergence of acquired mutations and alternative splicing of CD19 enables resistance to CART-19 Immunotherapy	2015	*Cancer Discovery*	Q1	24.373	251	Article	USA
Barrett DM	Chimeric antigen receptor therapy for cancer	2014	*Annual Review of Medicine*	Q1	14.97	197	Book Chapter	USA
Diak P	Tumor necrosis factor alpha blockers and malignancy in children forty-eight cases reported to the Food and Drug Administration	2010	*Arthritis and Rheumatism*	—	—	151	Article	USA
Fry TJ	CD22-targeted CAR T cells induce remission in B-ALL that is naive or resistant to CD19-targeted CAR immunotherapy	2018	*Nature Medicine*	Q1	32.621	123	Article	USA
Rodriguez- Galindo C	Treatment of Ewing sarcoma family of tumors: current status and outlook for the future	2003	*Medical and Pediatric Oncology*	—	—	129	Review	USA
Locatelli F	How I treat relapsed childhood acute lymphoblastic leukemia	2012	*Blood*	Q1	15.132	112	Review	Italy and Germany
Sugiyama H	Wilms’ tumor gene WT1: its oncogenic function and clinical application	2001	*International Journal of Hematology*	Q3	1.942	108	Review	Japan
Rosenfeld C	WT1 in acute leukemia, chronic myelogenous leukemia and myelodysplastic syndrome: therapeutic potential of WT1 targeted therapies	2003	*Leukemia*	Q1	10.023	103	Review	USA and Austria
Dagher R	Pilot trial of tumor-specific peptide vaccination and continuous infusion interleukin-2 in patients with recurrent Ewing sarcoma and alveolar rhabdomyosarcoma: an inter-institute NIH study	2002	*Medical and Pediatric Oncology*	—	—	102	Article	USA

^$^Data from the 2017 edition of Journal Citation Reports.

### Keyword Co-Occurrence Network Visualization

**Figure 2:**
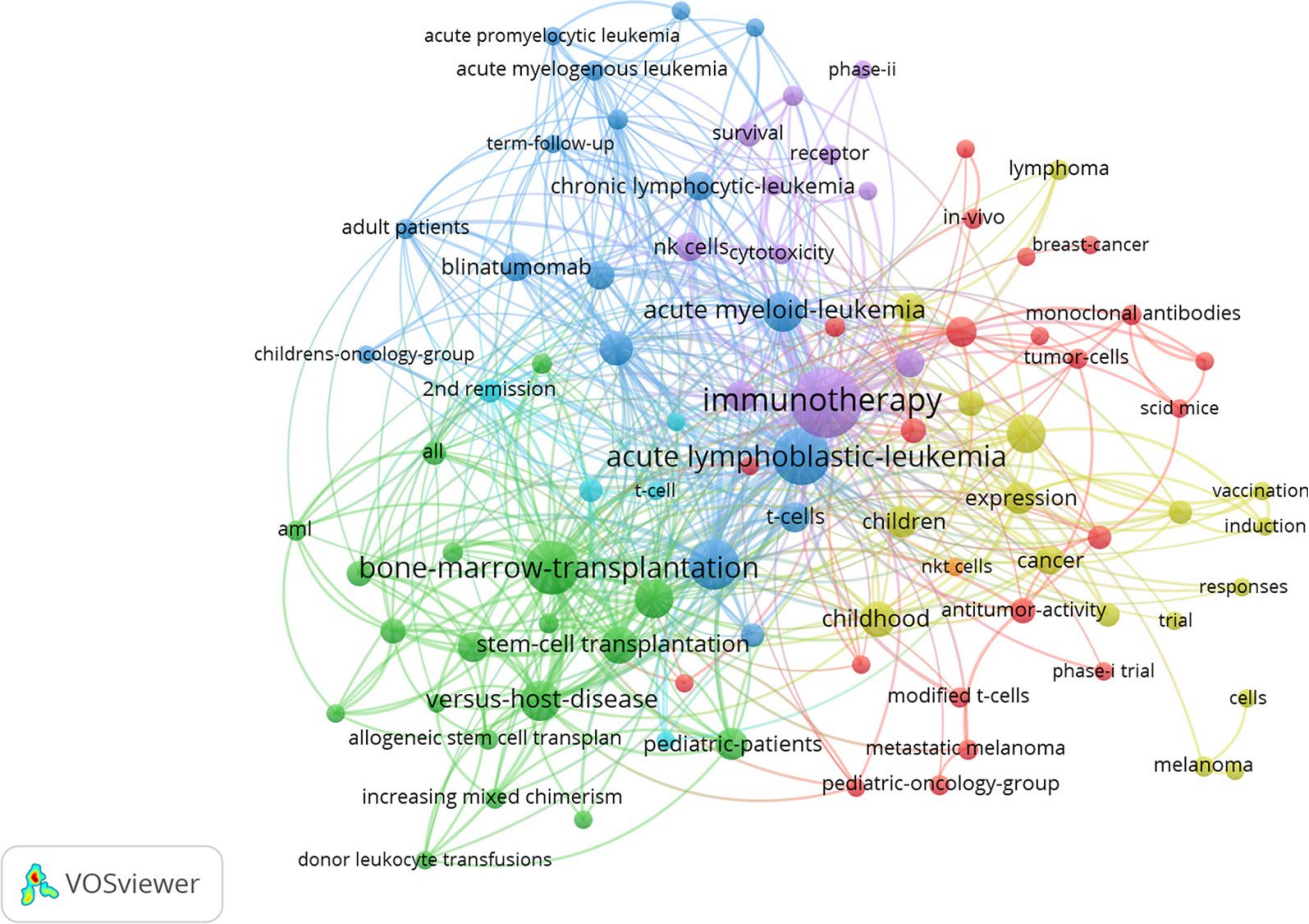
Keyword co-occurrence network visualization of the 100 most highly cited articles.

VOSviewer software was used to draw a figure for keyword co-occurrence network, setting the minimum number of occurrences at 3. As shown in **Figure 2**, circles representing keywords such as *acute lymphoblastic-leukemia*, *bone-marrow-transplantation*, *acute myeloid-leukemia*, *versus-host-disease*, and *stem cell transplantation* are larger than others, indicating that these keywords appeared more frequently. Blinatumomab, a BiTE drug, has been extensively studied.

### Researcher Coauthored Network Visualization

**Figure 3:**
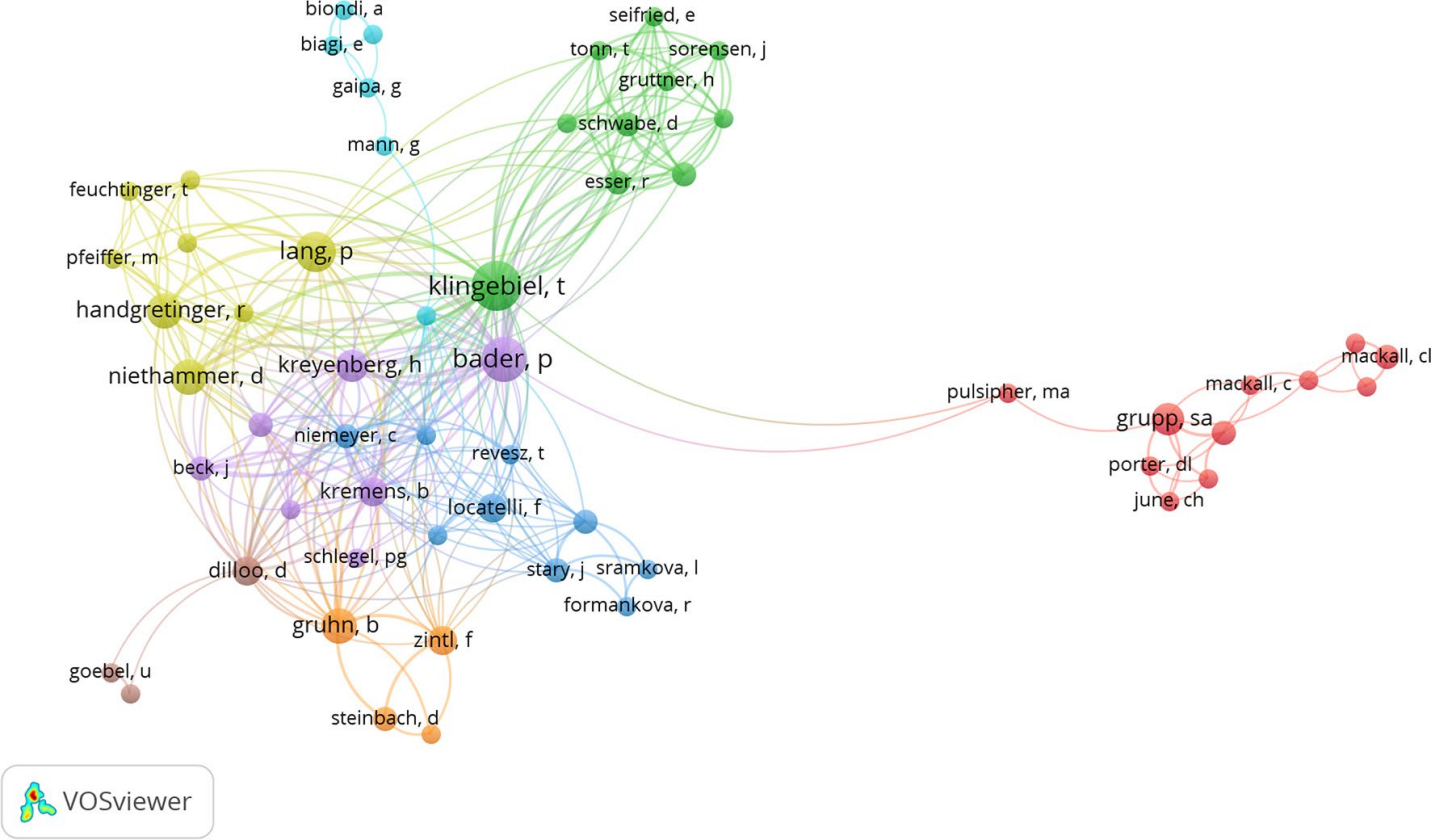
Author coauthorship network visualization of the 100 most highly cited articles.

VOSviewer software was used to analyze coauthorship network of authors, with the minimum number of coauthors at 2, and figure for coauthorship network was drawn (**Figure 3**). Accordingly, circles representing T. Klingebiel, P. Bader, and P. Lang are larger than others, and connection lines between them were denser and thicker, indicating that they contributed to more collaborating articles and had closer relation.

## Discussion

In this study, we identified and analyzed the top 100 highly cited articles in the field of immunotherapy for childhood leukemia. Through bibliometric analysis, the status and characteristics of publications in this field were explored, including publication journals, research institutions, authors, and other information. The trends of the most frequently cited articles in this field have been clarified, which provided ideas and directions for researchers.

According to publication years of the top 100 highly cited articles, every year could see two to nine articles highly cited, and only one-fourth of those articles were published in the past 5 years. Perhaps because immunotherapy is an emerging approach, a large amount of preclinical and clinical researches are still in progress. Among the top 100 articles, most were from European and American countries, few from Asia. Reason for such a phenomenon possibly is that leukemia has higher incidence in Europe and the United States. According to statistics, the risk of leukemia is 10 to 20 times higher in Europe and the United States than in Asia (Yang et al., 2015). Therefore, many research institutions in Europe and the United States have been exploring in this field. According to the number of citations, the most frequently cited articles in the top 100 ones were cited 471 times. Compared with other features (Liao et al., 2016; Wang et al., 2019), such figure was not large, probably because research in this field is still in the initial stage, and our research topic involved only the blood system and immunotherapy. However, considering physical, psychological, and financial burden from leukemia on the patients (Bosshard et al., 2018) and enormous potential of immunotherapy in treating this disease, researches in this area are important.

A total of 52 journals were involved in this study. The journals were arranged in descending order according to the number of the top 100 highly cited articles they published. The journals were divided into three groups, each with the same number of the articles, and then the number of journals in the three groups was 4, 14, and 34, respectively, approximate to 1:3^1^:3^2^. The distribution of these publications was consistent with Bradford’s Law (Bradford, 1985). Of the top 100 highly cited articles, 66% were published in Q1 (2017 edition of the journal citation report), 9% in Q2 and Q3, separately, and 5% in Q4. Most of them were published in journals possessing high impact factor, while these journals are often subscribed by more researchers, and high-quality research results face more opportunities to be cited (Callaham et al., 2002).

According to the results of keyword co-occurrence, the top 100 highly cited articles covered various aspects of immunotherapy for childhood leukemia, such as leukemia type, immunotherapy type, immunotherapy mechanism (Foster and Maude, 2018), immunotherapy experiments *in vitro*, animal experiments *in vitro* (McGinn et al., 2017; Hicks et al., 2019), preclinical studies, stem cell transplantation, changes in survival time after immunotherapy, and leukemia recurrence. According to the figure for keywords co-occurrence network, we can intuitively observe links between keywords and analyze hot topics of the researches. In recent years, a variety of immunotherapies have been approved for clinical leukemia treatment (Kantarjian et al., 2017; Kantarjian et al., 2018; Mueller et al., 2018; Jabbour et al., 2019). Growing preclinical studies have also been used to explore new immunotherapies for different types of leukemia.

From the figure for coauthorship network, coauthorship between authors and comparisons on the number of published articles can be visually observed. After multiple times of drawing network figure, the minimum times for author appearing was set at 2. Even so, most researchers were not presented in network diagram because they did not meet the conditions. Researchers in this field are scattered, and only few of them have published more articles. Researchers such as T. Klingebiel, P. Bader, P. Lang, and B. Gruhn from Germany were in close contact with other researchers in this field and have published more articles.

This study still had some limitations. On the one hand, some of the influential articles may be omitted. We only searched the ISI Web of Science core collection database, and articles in other sources such as PubMed and Scopus might be missed, so our final results may be affected by such operation (Riggs et al., 2017). On the other hand, the number of citations is only an index to evaluate academic influence of articles. Higher impact factors of journals, greater academic influence of authors in this field, Open Access publishing, higher visibility of institutions, and long publication time of literature may all have positive impact on the number of citations for articles (Calver and Bradley, 2010; Zhang and Poucke, 2017). Besides, the quality of the top 100 articles was not assessed, so the quality of the documents may be different. This may affect the interpretation of the results.

## Conclusions

In this study, we analyzed the top 100 highly cited articles on immunotherapy for childhood leukemia *via* bibliometrics. Most of the articles were published after the year 2000 on top journals in the field of oncology and hematology. Adoptive immunotherapy and stem cell transplantation are the main topics in these articles, which contributed to the development and optimization of immunotherapy. The United States and European countries such as Germany and Italy were major original countries in this research field. Professor T. Klingebiel from Goethe University Frankfurt represented a leader in the field of immunotherapy for childhood leukemia. This report provided insights into the features and research hotspots of highly cited articles on immunotherapy for childhood leukemia.

## Data Availability

All datasets generated for this study are included in the manuscript/**Supplementary Files**.

## Author Contributions

Y-HJ designed this study. QZ and B-HL performed search and collected data. Q-QZ re-checked data. Z-MZ performed analysis, Z-HZ rechecked. QZ wrote the manuscript. Y-HJ reviewed the manuscript.

## Conflict of Interest Statement

The authors declare that the research was conducted in the absence of any commercial or financial relationships that could be construed as a potential conflict of interest.
