# Supplementary material for: The Top 100 Highly Cited Original Articles on Immunotherapy for Childhood Leukemia
Source: Front Pharmacol. 2019 Sep 24;10:1100. doi: 10.3389/fphar.2019.01100 (PMC6769078; doi:10.3389/fphar.2019.01100)
Supplement: Supplementary file 1 [file Table_1.docx]

***Supplementary Material***

**The Top-100 Highly Cited Original Articles on Immunotherapy for Childhood Leukemia**

**Qing Zhong^1^, Bing-Hui Li^2,3^, Qi-Qi Zhu^1^, Zhi-Min Zhang^1^, Zhi-Hao Zou^1^, Ying-Hui Jin^1,2*^**

**^*^** **Correspondence:** Ying-Hui Jin: E-mail: jinyinghuiebm@163.com

**Supplementary TABLE S1** List of The top-100 highly cited original articles on Immunotherapy for Childhood Leukemia ranked according to their total citation counts^*^

| **Rank** | **Title** | **Journal** | **Impact factor** | **Total citation count** |
| --- | --- | --- | --- | --- |
| 1 | Biology, Risk Stratification, and Therapy of Pediatric Acute Leukemias: An Update | Journal of clinical oncology | 26.36 | 471 |
| 2 | Convergence of Acquired Mutations and Alternative Splicing of CD19 Enables Resistance to CART-19 Immunotherapy | Cancer discovery | 24.373 | 251 |
| 3 | Chimeric Antigen Receptor Therapy for Cancer | Annual review of medicine, vol 65 | 14.97 | 197 |
| 4 | Tumor Necrosis Factor alpha Blockers and Malignancy in Children Forty-Eight Cases Reported to the Food and Drug Administration | Arthritis and rheumatism | / | 151 |
| 5 | Treatment of Ewing sarcoma family of tumors: Current status and outlook for the future | Medical and pediatric oncology | / | 129 |
| 6 | CD22-targeted CAR T cells induce remission in B-ALL that is naive or resistant to CD19-targeted CAR immunotherapy | Nature medicine | 32.621 | 123 |
| 7 | How I treat relapsed childhood acute lymphoblastic leukemia | Blood | 15.132 | 112 |
| 8 | Wilms' tumor gene WT1: Its oncogenic function and clinical application | International journal of hematology | 1.942 | 108 |
| 9 | WT1 in acute leukemia, chronic myelogenous leukemia and myelodysplastic syndrome: therapeutic potential of WT1 targeted therapies | Leukemia | 10.023 | 103 |
| 10 | Pilot trial of tumor-specific peptide vaccination and continuous infusion interleukin-2 in patients with recurrent Ewing sarcoma and alveolar rhabdomyosarcoma: An inter-institute NIH study | Medical and pediatric oncology | / | 102 |
| 11 | IL-2 activated NK cell immunotherapy of three children after haploidentical stem cell transplantation | Blood cells molecules and diseases | 1.836 | 99 |
| 12 | CD20 up-regulation in pediatric B-cell precursor acute lymphoblastic leukemia during induction treatment: setting the stage for anti-CD20 directed immunotherapy | Blood | 15.132 | 93 |
| 13 | Natural killer cell allorecognition of missing self in allogeneic hematopoietic transplantation: a tool for immunotherapy of leukemia | Current opinion in immunology | 7.932 | 89 |
| 14 | Clinical implications of PRAME gene expression in childhood acute myeloid leukemia | Cancer genetics and cytogenetics | / | 86 |
| 15 | 2B4 (CD244) Signaling by Recombinant Antigen-specific Chimeric Receptors Costimulates Natural Killer Cell Activation to Leukemia and Neuroblastoma Cells | Clinical cancer research | 10.199 | 84 |
| 16 | Ex vivo expansion of highly purified NK cells for immunotherapy after haploidentical stem cell transplantation in children | Klinische padiatrie | 0.698 | 83 |
| 17 | Identification of a set of seven genes for the monitoring of minimal residual disease in pediatric acute myeloid leukemia | Clinical cancer research | 10.199 | 82 |
| 18 | The Future Is Now: Chimeric Antigen Receptors as New Targeted Therapies for Childhood Cancer | Clinical cancer research | 10.199 | 81 |
| 19 | Sarcoplasmic calcium-binding protein is an EF-hand-type protein identified as a new shrimp allergen | Journal of allergy and clinical immunology | 13.258 | 80 |
| 20 | Long-term outcome after haploidentical stem cell transplantation in children | Blood cells molecules and diseases | 1.836 | 76 |
| 21 | Advanced Stage, Increased Lactate Dehydrogenase, and Primary Site, but Not Adolescent Age (>= 15 Years), Are Associated With an Increased Risk of Treatment Failure in Children and Adolescents With Mature B-Cell Non-Hodgkin's Lymphoma: Results of the FAB LMB 96 Study | Journal of clinical oncology | 26.36 | 74 |
| 22 | Increasing mixed chimerism defines a high-risk group of childhood acute myelogenous leukemia patients after allogeneic stem cell transplantation where pre-emptive immunotherapy may be effective | Bone marrow transplantation | 4.497 | 64 |
| 23 | Early memory phenotypes drive T cell proliferation in patients with pediatric malignancies | Science translational medicine | 16.71 | 64 |
| 24 | Generation of CD3(+)CD56(+) cytokine-induced killer cells and their in vitro cytotoxicity against pediatric cancer | International journal of hematology | 1.942 | 62 |
| 25 | Immunotoxins against CD19 and CD22 are effective in killing precursor-B acute lymphoblastic leukemia cells in vitro | Leukemia | 10.023 | 58 |
| 26 | Preemptive immunotherapy in childhood acute myeloid leukemia for patients showing evidence of mixed chimerism after allogeneic stem cell transplantation | Blood | 15.132 | 57 |
| 27 | Cord blood comprises antigen-experienced T cells specific for maternal minor histocompatibility antigen HA-1 | Blood | 15.132 | 56 |
| 28 | An update on the management of severe idiopathic aplastic anaemia in children | British journal of haematology | 5.128 | 56 |
| 29 | Donor leukocyte infusion after hematopoietic stem cell transplantation in patients with juvenile myelomonocytic leukemia | Leukemia | 10.023 | 55 |
| 30 | Detectable minimal residual disease before allogeneic hematopoietic stem cell transplantation predicts extremely poor prognosis in children with acute lymphoblastic leukemia | Pediatric blood & cancer | 2.646 | 55 |
| 31 | Infusions of Allogeneic Natural Killer Cells as Cancer Therapy | Clinical cancer research | 10.199 | 52 |
| 32 | Clinical scale isolation of T cell-depleted CD56(+) donor lymphocytes in children | Bone marrow transplantation | 4.497 | 52 |
| 33 | Immune-based therapies for childhood cancer | Nature reviews clinical oncology | 24.653 | 52 |
| 34 | Treatment of SCID/human B cell precursor ALL with anti-CD19 and anti-CD22 immunotoxins | Leukemia | 10.023 | 51 |
| 35 | Negative depletion of alpha/beta(+) T cells and of CD19+B lymphocytes: A novel frontier to optimize the effect of innate immunity in HLA-mismatched hematopoietic stem cell transplantation | Immunology letters | 2.436 | 51 |
| 36 | Immature CD34(+)CD19(-) progenitor/stem cells in TEL/AML1-positive acute lymphoblastic leukemia are genetically and functionally normal | Blood | 15.132 | 50 |
| 37 | Early lymphocyte recovery post-allogeneic hematopoietic stem cell transplantation is associated with significant graft-versus-leukemia effect without increase in graft-versus-host disease in pediatric acute lymphoblastic leukemia | Bone marrow transplantation | 4.497 | 49 |
| 38 | PRAME gene expression in childhood acute lymphoblastic leukemia | Cancer genetics and cytogenetics | / | 48 |
| 39 | Allogeneic Transplantation for Pediatric Acute Lymphoblastic Leukemia: The Emerging Role of Peritransplantation Minimal Residual Disease/Chimerism Monitoring and Novel Chemotherapeutic, Molecular, and Immune Approaches Aimed at Preventing Relapse | Biology of blood and marrow transplantation | 4.484 | 47 |
| 40 | IL-2 Stimulated but Not Unstimulated NK Cells Induce Selective Disappearance of Peripheral Blood Cells: Concomitant Results to a Phase I/II Study | Plos one | 2.766 | 45 |
| 41 | Evaluation of immunomodulatory treatment based on conventional and lineage-specific chimerism analysis in patients with myeloid malignancies after myeloablative allogeneic hematopoietic cell transplantation | Leukemia | 10.023 | 44 |
| 42 | Lysis of MYCN-amplified neuroblastoma cells by MYCN peptide-specific cytotoxic T lymphocytes | Cancer research | 9.13 | 44 |
| 43 | Haploidentical SCT in children: an update and future perspectives | Bone marrow transplantation | 4.497 | 43 |
| 44 | CD19-redirected chimeric antigen receptor-modified T cells: a promising immunotherapy for children and adults with B-cell acute lymphoblastic leukemia (ALL) | Therapeutic advances in hematology | / | 42 |
| 45 | Preemptive alloimmune intervention in high-risk pediatric acute lymphoblastic leukemia patients guided by minimal residual disease level before stem cell transplantation | Leukemia | 10.023 | 41 |
| 46 | CD1d expression on B-precursor acute lymphoblastic leukemia subsets with poor prognosis | Leukemia | 10.023 | 40 |
| 47 | An investigation into the potential use of serum Hsp70 as a novel tumour biomarker for Hsp90 inhibitors | Biomarkers | 1.976 | 40 |
| 48 | Novel agents for the treatment of childhood acute leukemia | Therapeutic advances in hematology | / | 39 |
| 49 | Novel associations between activating killer-cell immunoglobulin-like receptor genes and childhood leukemia | Blood | 15.132 | 39 |
| 50 | Harnessing T cells to fight cancer with BiTE (R) antibody constructs - past developments and future directions | Immunological reviews | 9.217 | 36 |
| 51 | Relationship between minimal residual disease measured by multiparametric flow cytometry prior to allogeneic hematopoietic stem cell transplantation and outcome in children with acute lymphoblastic leukemia | Haematologica-the hematology journal | / | 35 |
| 52 | Preliminary results of the safety of immunotherapy with gemtuzumab ozogamicin following reduced intensity allogeneic stem cell transplant in children with CD33(+) acute myeloid leukemia | Clinical cancer research | 10.199 | 35 |
| 53 | Targeting Human C-Type Lectin-like Molecule-1 (CLL1) with a Bispecific Antibody for Immunotherapy of Acute Myeloid Leukemia | Angewandte chemie-international edition | 12.102 | 34 |
| 54 | Human gamma delta Thymocytes Are Functionally Immature and Differentiate into Cytotoxic Type 1 Effector T Cells upon IL-2/IL-15 Signaling | Journal of immunology | 4.539 | 33 |
| 55 | Expansion of natural killer cells with lytic activity against autologous blasts from adult and pediatric acute lymphoid leukemia patients in complete hematologic remission | Haematologica-the hematology journal | / | 32 |
| 56 | Early recipient chimerism testing in the T- and NK-cell lineages for risk assessment of graft rejection in pediatric patients undergoing allogeneic stem cell transplantation | Leukemia | 10.023 | 32 |
| 57 | T-cell responses against CD19(+) pediatric acute lymphoblastic leukemia mediated by bispecific T-cell engager (BiTE) are regulated contrarily by PD-L1 and CD80/CD86 on leukemic blasts | Oncotarget | / | 31 |
| 58 | Generation of Tumor Antigen-Specific T Cell Lines from Pediatric Patients with Acute Lymphoblastic Leukemia-Implications for Immunotherapy | Clinical cancer research | 10.199 | 30 |
| 59 | IMC-EB10, an anti-FLT3 monoclonal antibody, prolongs survival and reduces nonobese diabetic/severe combined immunodeficient engraftment of some acute lymphoblastic leukemia cell lines and primary leukemic samples | Cancer research | 9.13 | 30 |
| 60 | Immune function in children under chemotherapy for standard risk acute lymphoblastic leukaemia - a prospective study of 20 paediatric patients | British journal of haematology | 5.128 | 30 |
| 61 | Harnessing the Immunotherapy Revolution for the Treatment of Childhood Cancers | Cancer cell | 22.844 | 30 |
| 62 | Children with myelodysplastic syndrome (MDS) and increasing mixed chimaerism after allogeneic stem cell transplantation have a poor outcome which can be improved by pre-emptive immunotherapy | British journal of haematology | 5.128 | 29 |
| 63 | Restricted Cell Surface Expression of Receptor Tyrosine Kinase ROR1 in Pediatric B-Lineage Acute Lymphoblastic Leukemia Suggests Targetability with Therapeutic Monoclonal Antibodies | Plos one | 2.766 | 29 |
| 64 | International Working Group consensus response evaluation criteria in lymphoma (RECIL 2017) | Annals of oncology | 13.93 | 28 |
| 65 | WT1 peptide vaccination following allogeneic stem cell transplantation in pediatric leukemic patients with high risk for relapse: successful maintenance of durable remission | Leukemia | 10.023 | 27 |
| 66 | Juvenile myelomonocytic leukemia: molecular pathogenesis informs current approaches to therapy and hematopoietic cell transplantation | Frontiers in pediatrics | 2.335 | 27 |
| 67 | Cellular kinetics of CTL019 in relapsed/refractory B-cell acute lymphoblastic leukemia and chronic lymphocytic leukemia | Blood | 15.132 | 26 |
| 68 | Alternative allogeneic donor sources for transplantation for childhood diseases: Unrelated cord blood and haploidentical family donors | Biology of blood and marrow transplantation | 4.484 | 26 |
| 69 | Novel targeted drug therapies I for the treatment of childhood acute leukemia | Expert review of hematology | 1.937 | 26 |
| 70 | Recent thymic emigrants and prognosis in T- and B-cell childhood hematopoietic malignancies | International journal of cancer | 7.36 | 26 |
| 71 | Clinical Cancer Advances 2018: Annual Report on Progress Against Cancer From the American Society of Clinical Oncology | Journal of clinical oncology | 26.36 | 25 |
| 72 | Risk factors and timing of relapse after allogeneic transplantation in pediatric ALL: for whom and when should interventions be tested? | Bone marrow transplantation | 4.497 | 25 |
| 73 | Approved CAR T cell therapies: ice bucket challenges on glaring safety risks and long-term impacts | Drug discovery today | 6.848 | 25 |
| 74 | CD40 activation of BCP-ALL cells generates 11L-10-producing, 11L-12-defective APCs that induce allogeneic T-cell anergy | Blood | 15.132 | 24 |
| 75 | Partially matched related donor peripheral blood progenitor cell transplantation in paediatric patients adding fludarabine and anti-lymphocyte gamma-globulin | Bone marrow transplantation | 4.497 | 24 |
| 76 | Reduced-Intensity Conditioning Regimens for Allogeneic Transplantation in Children with Acute Lymphoblastic Leukemia | Biology of blood and marrow transplantation | 4.484 | 24 |
| 77 | Advances in Chimeric Antigen Receptor Immunotherapy for Neuroblastoma | Discovery medicine | 2.398 | 24 |
| 78 | WT1 (WILMS TUMOR 1) PEPTIDE IMMUNOTHERAPY FOR CHILDHOOD RHABDOMYOSARCOMA: A Case Report | Pediatric hematology and oncology | 1.154 | 23 |
| 79 | Identification of candidate target antigens for antibody-based immunotherapy in childhood B-cell precursor ALL | Klinische padiatrie | 0.698 | 22 |
| 80 | The CD70/CD27 Pathway Is Critical for Stimulation of an Effective Cytotoxic T Cell Response against B Cell Precursor Acute Lymphoblastic Leukemia | Journal of immunology | 4.539 | 21 |
| 81 | Relapse of childhood ALL, AML and MDS after allogeneic stem cell transplantation can be prevented by donor lymphocyte infusion in a critical stage of increasing mixed chimerism | Klinische padiatrie | 0.698 | 21 |
| 82 | Extracorporeal photopheresis (photochemotherapy) in the treatment of acute and chronic graft versus host disease: immunological mechanisms and the results from clinical studies | Cancer immunology immunotherapy | 4.225 | 21 |
| 83 | On the use of donor-derived iNKT cells for adoptive immunotherapy to prevent leukemia recurrence in pediatric recipients of HLA haploidentical HSCT for hematological malignancies | Clinical immunology | 3.557 | 20 |
| 84 | gamma delta T cell-meciated antibody-dependent cellular cytotoxicity with CD19 antibodies assessed by an impedance-based label-free real-time cytotoxicity assay | Frontiers in immunology | 5.511 | 20 |
| 85 | Chimerism-directed adoptive immunotherapy in the prevention and treatment of post-transplant relapse of leukemia in childhood | Haematologica | 9.09 | 20 |
| 86 | Clinical overview of anti-CD19 BiTE (R) and ex vivo data from anti-CD33 BiTE (R) as examples for retargeting T cells in hematologic malignancies | Molecular immunology | 3.188 | 20 |
| 87 | The only proposed T-cell epitope derived from the TEL-AML1 translocation is not naturally processed | Blood | 15.132 | 19 |
| 88 | The role of stem cell transplantation for chronic myelogenous leukemia in the 21st century | Blood | 15.132 | 19 |
| 89 | Therapeutic opportunities for counteracting apoptosis resistance in childhood leukaemia | British journal of haematology | 5.128 | 19 |
| 90 | WT1 Peptide Immunotherapy for Cancer in Children and Young Adults | Pediatric blood & cancer | 2.646 | 19 |
| 91 | Cancer Antigen WT1 Protein-Derived Peptide-Based Treatment of Cancer - Toward the Further Development | Current medicinal chemistry | 3.469 | 19 |
| 92 | Update in childhood acute myeloid leukemia: recent developments in the molecular basis of disease and novel therapies | Current opinion in hematology | 2.821 | 19 |
| 93 | Safety and tolerability of allogeneic dendritic cell vaccination with induction of Wilms tumor 1-specific T cells in a pediatric donor and pediatric patient with relapsed leukemia: a case report and review of the literature | Cytotherapy | 3.993 | 19 |
| 94 | Anti-CD19 and anti-CD22 monoclonal antibodies increase the effectiveness of chemotherapy in Pre-B acute lymphoblastic leukemia cell lines | Leukemia research | 2.319 | 19 |
| 95 | FDA Approval Summary: Tocilizumab for Treatment of Chimeric Antigen Receptor T Cell-Induced Severe or Life-Threatening Cytokine Release Syndrome | Oncologist | 5.306 | 19 |
| 96 | CD40 ligand-stimulated B cell precursor leukemic cells elicit interferon-gamma production by autologous bone marrow T cells in childhood acute lymphoblastic leukemia | Leukemia | 10.023 | 18 |
| 97 | Progress in chimerism analysis in childhood malignancies - the dilemma of biostatistical considerations and ethical implications | Leukemia | 10.023 | 18 |
| 98 | Children's Cancer Group trials of interleukin-2 therapy to prevent relapse of acute myelogenous leukemia | Cancer journal from scientific american | / | 18 |
| 99 | Vaccine therapies for pediatric malignancies | Cancer journal | 3.519 | 17 |
| 100 | A review of blinatumomab, a novel immunotherapy | Journal of oncology pharmacy practice | 1.908 | 17 |

^*^Total citation count update to June 15, 2019; the impact factor data from the 2017 edition of Journal Citation Reports.
